# Meta-Analysis of Randomized Controlled Trials Comparing EX-PRESS Implantation with Trabeculectomy for Open-Angle Glaucoma

**DOI:** 10.1371/journal.pone.0100578

**Published:** 2014-06-27

**Authors:** Wei Wang, Xiulan Zhang

**Affiliations:** Zhongshan Ophthalmic Center, State Key Laboratory of Ophthalmology, Sun Yat-Sen University, Guangzhou, People's Republic of China; Bascom Palmer Eye Institute, University of Miami School of Medicine, United States of America

## Abstract

**Purpose:**

To evaluate the efficacy and safety of EX-PRESS implantation compared with trabeculectomy for uncontrolled open-angle glaucoma.

**Methods:**

Pertinent randomized controlled trials were identified through systematic searches of the PubMed, EMBASE, and Cochrane Library. The efficacy measures utilized were the weighted mean differences (WMDs) for the intraocular pressure reduction (IOPR), the reduction in glaucoma medications, the change of visual acuity, and the relative risks (RRs) for operative success rates. The safety measures utilized were RRs for postoperative complications. The pooled effects were calculated using the random-effects model.

**Results:**

Four randomized controlled trials of 292 eyes were included in this meta-analysis. The WMDs of the IOPR comparing the EX-PRESS with trabeculectomy were −0.25 (95% Cl: −3.61 to 3.11) at 6 month, 0.053 (−4.31 to 4.42) at 12 months, 0.81 (−4.06 to 5.67) at 24 months, and 0.20 (−2.11 to 2.51) at final follow-up. There was no statistically significance for IOPR at any point after surgery. There were also no significant differences in the reduction in glaucoma medications or visual acuity between the groups. The pooled relative risks comparing EX-PRESS with Trabeculectomy were 1.36 (1.11 to 1.66) for the complete operative success rate and 1.05 (0.94 to 1.17) for the qualified operative success rate. EX-PRESS and Trabeculectomy were associated with similar incidences in most complications with the exception of hyphema, with pooled RR being 0.18 (0.046 to 0.66).

**Conclusions:**

EX-PRESS implantation and trabeculectomy have similar efficacy in IOP-lowering, medication reduction, vision recovery, and qualified operative success rates. EX-PRESS associated with higher rates of complete operative success and fewer hyphema than with Trabeculectomy. However, these should be interpreted with caution because of the inherent limitations of the included studies.

## Introduction

The EX-PRESS implant, one of the newest modifications in glaucoma filtration surgery, is a nonvalved stainless steel tube currently available in 3 models (X, P, and R models) and is already approved and widely used in Europe, the US, and Japan [1]. Originally, it was inserted at the limbus directly under the conjunctiva to connect the anterior chamber space directly to the subconjunctival space. This procedure was associated with high hypotony rates and erosion of the device through the conjunctiva, which led to the current technique of implantation under a partial thickness sclera flap, providing resistance to aqueous outflow and reducing the risk for conjunctival erosion [2]. Unlike a standard trabeculectomy, the EX-PRESS implantation does not require the traditional sclerectomy or an iridectomy.

To date, several retrospective and prospective studies have compared the safety and efficacy of the EX-PRESS implant under a scleral flap with standard trabeculectomy and have reported conflicting results [3]–[15]. Previously, we conducted a first meta-analysis of 2 randomized controlled trials (RCTs) and 6 non-randomized comparative studies, and the results showed that EX-PRESS was associated with equivalent efficacy to Trabeculectomy in lowering IOP [16]. There were also comparable proportions of patients who reached the IOP target with EX-PRESS and Trabeculectomy [16]. However, these results have not been supported by 2 new RCTs [7], [8]. Thus, an additional meta-analysis of this topic is warranted. Our objective was to perform a meta-analysis on RCTs comparing EX-PRESS and Trabeculectomy in the treatment of uncontrolled open-angle glaucoma (OAG).

## Materials and Methods

This meta-analysis was performed according to a predetermined protocol described in the next paragraph, and the PRISMA (Preferred Reporting Items for Systematic Review and Meta-Analyses) statement was followed in all stages of the process [17] (Checklist S1).

### 1. Literature search

Clinical trials were identified through a systematic search consisting of (1) an electronic search of PubMed, EMBASE, and the Cochrane Library; (2) manual searches of the reference lists of original reports and review articles retrieved through the electronic searches; and (3) extensive Internet searches, including websites of professional associations and the Google Scholar search engine. The following terms, adapted for each database, were used for the searches: EX-PRESS, glaucoma, and trabeculectomy. No language or date restrictions were applied. The computerized searches covered the period from inception to December 2013.

### 2. Inclusion and exclusion criteria

The articles were considered eligible if the studies met the following inclusion criteria: (1) study type: RCTs; (2) population: OAG patients (but not including normal-tension glaucoma or ocular hypertension) who failed to respond to conservative therapy; (3) intervention: EX-PRESS versus Trabeculectomy; (4) outcome variables: at least 1 of the outcomes of interest was included; (5) and a follow-up time of at least 6 months. Abstracts from conferences and full texts without raw data available for retrieval, duplicate publications, letters, and reviews were excluded. For publications reporting on the same study population, the most informative article was included, and data that could not be obtained from this publication were obtained from others.

### 3. Data extraction

Data were extracted from each RCT by 2 independent reviewers (W.W. and X.Z.). Any discrepancies between the 2 independent data extractions were resolved by discussion to reach a consensus. For the eligible studies, the following data were extracted: (1) general characteristics (title, first author, journal title, and year of publication); (2) methodology (type of study, country of origin, sequence generation, allocation concealment, masking or blinding, incomplete outcome data, selective reporting, and other sources of bias); (3) subjects (recruitment site, enrollment periods, inclusion criteria, exclusion criteria, and general patient characteristics); (4) interventions (concentration of MMC and exposure time); (5) outcomes (measurement, follow-up time and loss of follow-up); (6) analysis (statistical methods); and (7) results (quantitative results and qualitative results).

### 4. Assessment of methodology quality

The methodological quality of each study was assessed using the risk-of-bias tool outlined in the Cochrane Handbook for Systematic Reviews of Interventions (version 5.1.0) [18]. Two reviewers (W.W. and X.Z.) subjectively reviewed all studies and assessed 6 different key aspects that influence the quality of an RCT, including sequence generation, allocation concealment, blinding of participants and outcome assessors, management of eventual incomplete outcome data, completeness of outcome reporting, and other potential threats to validity.

### 5. Outcome Measures

The primary outcome was the IOP reduction (IOPR) from preoperative to postoperative status. The secondary outcome measure was the difference in the reduction in glaucoma medications and the change of visual acuity. For efficacy, the proportion of complete operative success and qualified operative success was also used. Complete operative success was defined as the target endpoint IOP without medications, and qualified operative success was defined as the target endpoint IOP with or without medications. The outcomes of safety were complication rates in either group, including hypotony, choroidal effusion, flat anterior chamber, hyphema, bleb leak, and at least 1 complication per eye.

### 6. Statistical Analysis

All analyses were performed on an intent-to-treat basis (i.e., all patients assigned randomly to a treatment group were included in the analyses according to the assigned treatment, irrespective of whether they received treatment or were excluded from analysis by the study investigators). The data from individual studies were pooled by using the random-effect model with the DerSimonian-Laird method, which considers within-study and between-study variations [19]. For continuous variables, the weighted mean differences (WMDs) were measured, while the relative risks (RRs) were measured for dichotomous variables. All outcomes were reported with a 95% confidence interval (CI). Statistical heterogeneity among studies was evaluated with the χ2 and I^2^ tests [20]. P<0.05 was considered statistically significant on the test for overall effect. One-way sensitivity analyses were performed by iteratively removing 1 study at a time to assess the stability of the meta-analysis results. Only outcomes of interest that were reported in ≥3 studies were included in the sensitivity analysis. Potential publication bias was estimated by both visually evaluating a funnel plot and the Egger test. All statistical analyses were conducted using the Stata software package (version 12.0; Stata Corp., College Station, TX).

## Results

### 1. Literature search

**Figure 1:**
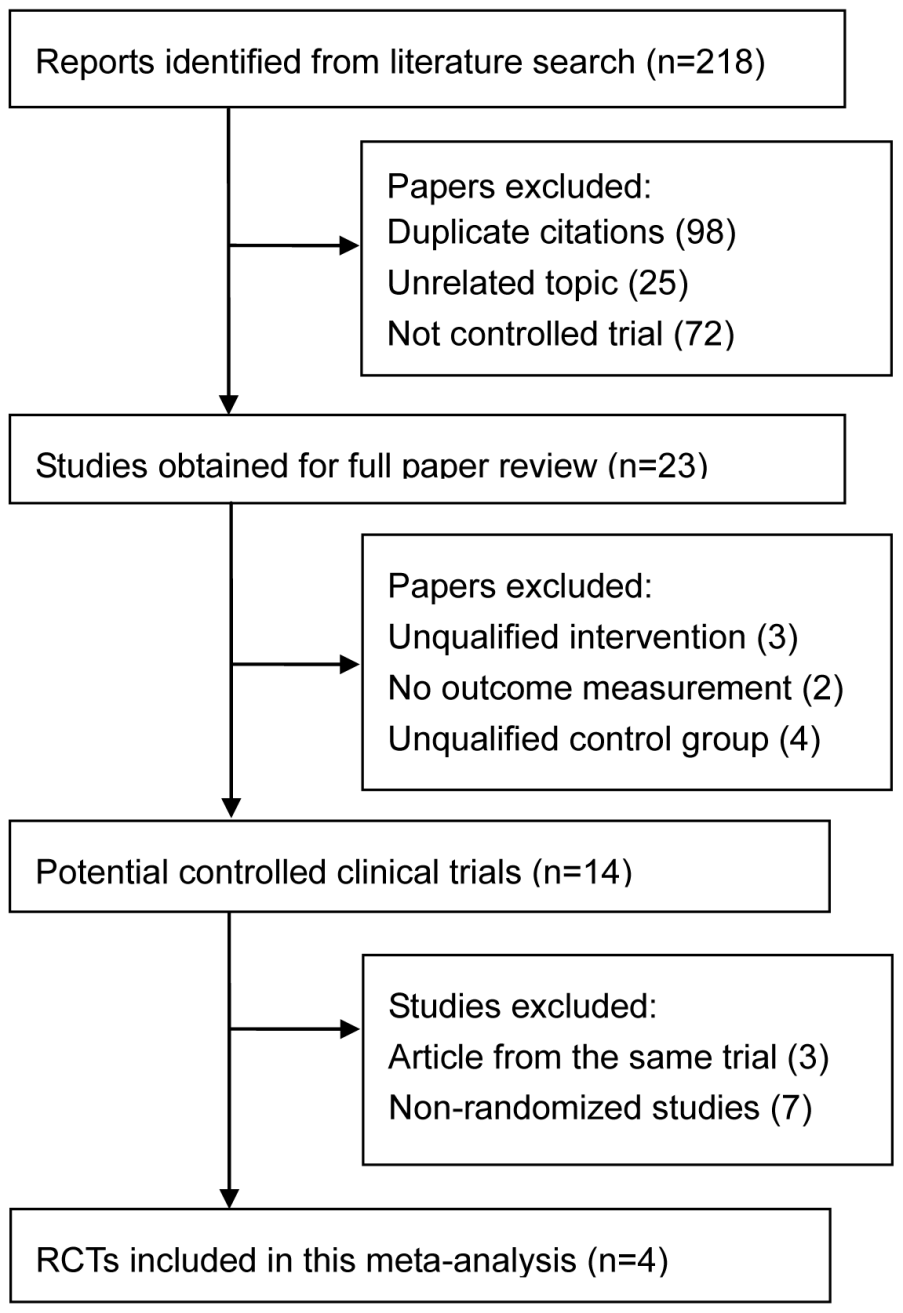
Flow of RCTs included in the meta-analysis. RCTs indicates randomized controlled trials.

The selection of studies is summarized in Figure 1. A total of 218 articles were initially identified. The abstracts were reviewed, and 23 articles with potentially relevant trials were reviewed in their entirety. Subsequently, 14 articles with full texts that potentially met the inclusion criteria were assessed [3]–[14]. Of these 14 articles, 7 [3]–[6], [11], [12] were not randomized studies, and 3 [13], [14], [21] represented duplicate data. Hence, a final total of 4 RCTs [7]–[10] were included in this meta-analysis.

### 2. Characteristics and Quality of Eligible Studies

The RCTs were published between 2011 and 2013, which involved a total of 292 eyes (146 in the EX-PRESS group and 146 in the Trabeculectomy group). The characteristics of the eligible studies are summarized in Table 1. Two studies [7], [9] only included patients with primary open angle glaucoma (POAG), and 2 studies [8], [10] included patients with POAG, pseudoexfoliation glaucoma, and pigmentary glaucoma. The studies were done in the USA, the Netherlands, South Africa, and Canada. The mean age of the patients ranged from 61.9 to 69.4 years, and the percentage of male patients ranged from 47.5% to 68.0%. Sample sizes in these studies ranged from 30 to 120. The mean follow-up period ranged from 12 to 66.4 months. Success was defined as a target endpoint of IOP≤18 mm Hg in all RCTs. The agreement between the 2 reviewers' quality assessment of the trials was scored by the κ coefficient (a measure of agreement), which was 0.85, with 92.1% observed agreement. The risk of bias in the RCTs is shown in Table 2. In general, the included trials were at low risk of bias for most of the aspects evaluated. All 4 RCTs described the specific methods of random sequence generation. Allocation concealment was described for 1 study. Information about the blindness assessment was not described in all articles. Adequate assessments of each outcome and selective outcome reporting avoided were all reported in the RCTs. All 4 RCTs adopted intention-to-treat analysis and were free of other biases.

**Table 1:** Characteristics of RCTs Comparing EX-PRESS implantation With Trabeculectomy.

Author(year)	Location	Center	Group	Eyes	Patients	Caucasian	Male(%)	Age(mean±SD)	Follow-up(mo)
de Jong(2011)	Dutch	Single	EX-PRESS	39	39	84.60%	47.50%	62.4±14.7	65.6(62.8–68.5)
			Trab	39	39	92.30%	67.50%	68.6±11.5	66.4(62.8–78.5)
Dahan(2012)	South Africa	Single	EX-PRESS	15	15	20.00%	66.67%	65.4±13.7	23.6±6.9
			Trab	15	15	20.00%	66.67%	65.4±13.7	23.6±6.9
Beltran-Agullo (2013)	Canada	Single	EX-PRESS	33	33	70.00%	68.00%	61.9±13.9	12
			Trab	31	31	45.00%	61.00%	65.9±11.5	12
Netland(2013)	USA Canada	Multiple	EX-PRESS	59	59	42.40%	54.20%	69.4±11.6	24
			Trab	61	61	32.80%	54.10%	67.8±10.4	24

RCTs indicates randomized controlled trials; mo, months.

**Table 2:** Evaluation of the risks of bias of RCTs included in the meta-analysis.

First author (year)	Sequence Generation	Allocation Concealment	Blinding	Adequate assessment of each outcome	Selective reporting avoided	No Other Bias
de Jong(2011)	Yes	Unclear	No	Yes	Yes	Yes
Dahan(2012)	Yes	Yes	No	Yes	Yes	Yes
Beltran-Agullo(2013)	Yes	Unclear	No	Yes	Yes	Yes
Netland(2013)	Yes	Unclear	No	Yes	Yes	Yes

RCTs indicates randomized controlled trials.

### 3. Efficacy analysis

**Figure 2:**
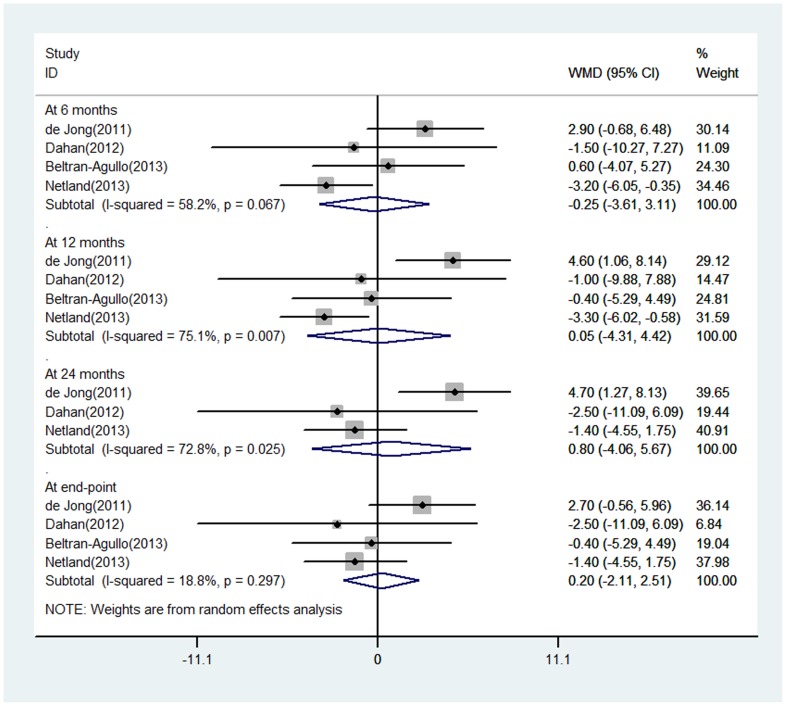
Forest plot showing meta-analysis of intraocular pressure reduction in randomized trials comparing EX-PRESS implantation with trabeculectomy. WMD indicates weighted mean difference, which was computed by using a random effects model.

Four studies reported the IOPR at various time points, 4 of them at 6 months and 12 months, 3 at 24 months, and 4 at final follow-up. The IOP reduction was numerically larger for the EX-PRESS group at all intervals with the exception of 6 months. When comparing the EX-PRESS group with the Trabeculectomy group, the WMDs of the IOPR were −0.25 (95% Cl: −3.61 to 3.11) at 6 months, 0.053 (−4.31 to 4.42) at 12 months, 0.81 (−4.06 to 5.67) at 24 months, and 0.20 (−2.11 to 2.51) at final follow-up. There was substantial statistical heterogeneity in these analyses, and the differences in IOPR were all not statistically significant (Figure 2).

**Figure 3:**
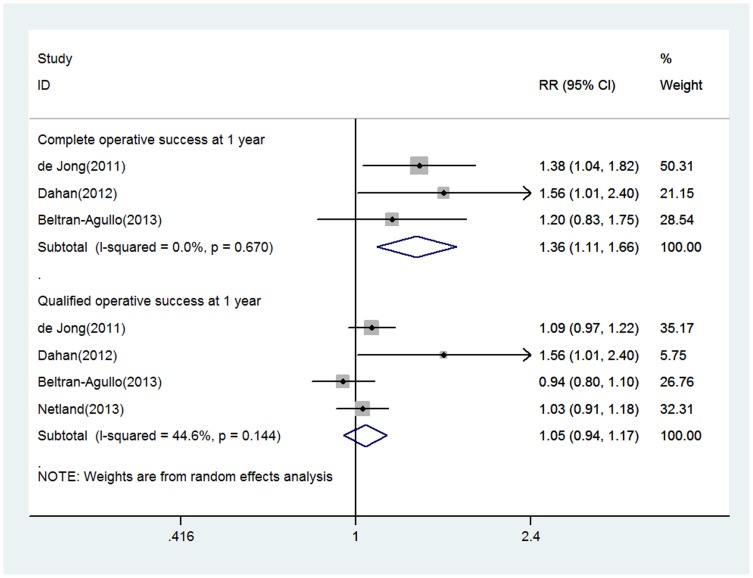
Forest plot showing meta-analysis of operative success rate in randomized trials comparing EX-PRESS implantation with trabeculectomy. RR indicates relative risks, which was computed by using a random effects model.

Concerning the success rate, 3 studies reported the probability of complete operative success at 1 year, and EX-PRESS was associated with higher complete operative success rates compared with Trabeculectomy, with the pooled RR being 1.35 (1.11 to 1.66) (Figure 3). Four studies reported the proportion of patients achieving qualified operative success at 1 year, and no significant difference was found, with the pooled RR being 1.05 (0.94 to 1.17) (Figure 3). There was no statistical evidence of heterogeneity across these studies (both P values>0.10; I^2^<50%).

**Figure 4:**
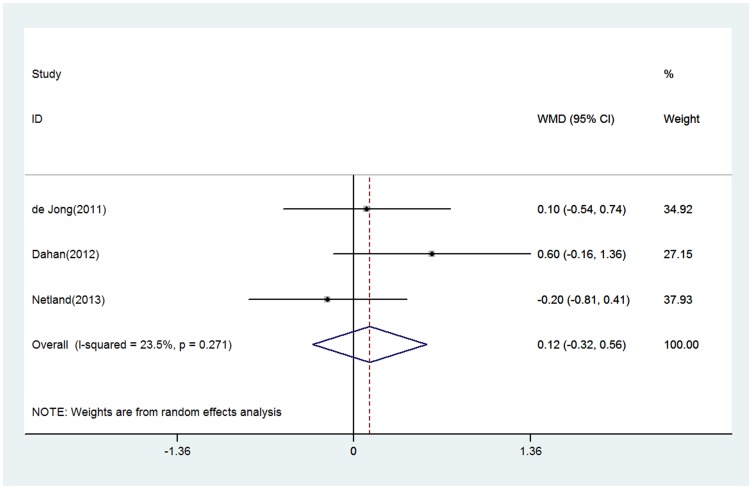
Forest plot showing meta-analysis of glaucoma medication reduction in randomized trials comparing EX-PRESS implantation with trabeculectomy. WMD indicates weighted mean difference, which was computed by using a random effects model.

With respect to glaucoma medication reduction, there was no significant difference between the 2 groups (Figure 4). The WMDs of reductions in the number of glaucoma medications after surgery (95% CI) were −0.12 (−0.32 to 0.56). No evidence of heterogeneity was observed (P = 0.27; I^2^ = 23.5%). No contradictory or significant differences were observed in the results of the sensitivity analysis compared to the previous analysis.

**Figure 5:**
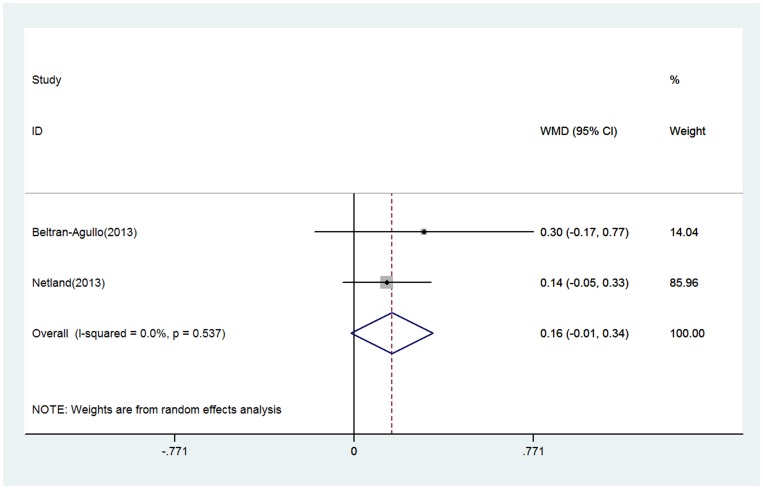
Forest plot showing meta-analysis of the change of visual acuity in randomized trials comparing EX-PRESS implantation with trabeculectomy. WMD indicates weighted mean difference, which was computed by using a random effects model.

In respect to visual rehabilitation, 2 RCTs reported data on changes in visual acuity from baseline to endpoint. Pooled results showed similar visual recovery between the 2 groups, with WMD being 0.16 (−0.014 to 0.34) (Figure 5). Two studies showed that visual acuity returning to baseline vision was more rapid in the EX-PRESS group. However, we did not perform a meta-analysis because the reports lacked a uniform standard of measuring the restore speed of visual acuity.

### 4. Safety analysis

Adverse events in the RCTs comparing EX-PRESS and Trabeculectomy are shown in Table 3. Flat anterior chamber and hyphema were 2 of the most commonly reported postoperative complications. EX-PRESS was associated with a significantly lower frequency of hyphema than Trabeculectomy, with a pooled RR of 0.18 (0.05 to 0.66). Numerically lower but nonsignificant proportions of EX-PRESS patients than Trabeculectomy patients had other postoperative complications, such as hypotony, choroidal effusion, flat anterior chamber, bleb leak, and at least 1 complication per eye, with the pooled RRs being 0.61 (0.14 to 2.64), 0.71 (0.30 to 1.71), 0.91 (0.43 to1.93), 0.98 (0.35 to 2.75), and 0.83 (0.45 to 1.53), respectively.

**Table 3:** Postoperative complications Comparing EX-PRESS implantation With Trabeculectomy.

Complication	Studies(n)	RR (95% CI)			Test for Heterogeneity				Test for Overall Effect	
		Estimate	ll	ul	ϰ2	df	P	I^2^	Z	P
Hypotony	2	0.61	0.14	2.64	2.29	1	0.131	56.30%	0.66	0.508
Choroidal effusion	3	0.71	0.30	1.71	1.96	2	0.375	0.00%	0.76	0.446
Flat anterior chamber	4	0.91	0.43	1.93	1.83	3	0.609	0.00%	0.26	0.796
Hyphema	4	0.18	0.05	0.66	0.47	3	0.926	0.00%	2.57	**0.010**
Bleb leak	4	0.98	0.35	2.75	1.13	3	0.770	0.00%	0.03	0.972
At least 1 complication	3	0.83	0.45	1.53	4.89	2	0.087	59.10%	0.59	0.558

RR indicates relative risks, which were computed by using a random effects model. 95% CI indicates 95% confidence interval.

### 5. Sensitivity Analysis and Publication Bias

To evaluate the robustness of the results, each study in the meta-analysis was excluded in turn to reflect the influence of individual studies on the pooled estimates of IOPR at every time point. The results indicated that the random-effect estimates before or after the deletion of any single study were generally similar, suggesting high stability in the meta-analysis results (data not shown). Due to the limited number (<10) of studies included in each analysis, publication bias was not assessed.

## Discussion

Glaucoma is the leading cause of irreversible blindness worldwide and represents a significant public health concern [22]. Despite the introduction of various modern and minimally invasive glaucoma procedures, trabeculectomy remains the standard surgical procedure for this potentially blinding condition [23]. However, trabeculectomy is associated with significant complications, such as choroidal detachment, hyphema, malignant glaucoma, and endophthalmitis. EX-PRESS was developed to improve the long-term surgical success of trabeculectomy but with fewer complications [1].

In this study, we found a similar mean IOP change from the baseline IOP and qualified operative success rates in the EX-PRESS and trabeculectomy groups, which is in agreement with our previous meta-analysis [16]. The number of glaucoma medications used was not significantly different in the EX-PRESS group compared with the trabeculectomy group. Subjects treated with the EX-PRESS implant, compared with trabeculectomy, had higher complete operative success during the first year. As for post-operative complications, the lack of significant difference in most complications except hyphema was noted. EX-PRESS appears to provide at least a comparable efficacy and safety profile when compared to trabeculectomy. Sensitivity analysis suggested that the results were robust.

The EX-PRESS implant is a nonvalved device that shunts aqueous outflow from the anterior chamber into the subconjunctival space adjacent to the limbus. In contrast with trabeculectomy, EX-PRESS implantation does not require a sclerostomy or peripheral iridotomy [24]. This may reduce intraoperative time, decrease postoperative inflammation, reduce the likelihood of hyphema, and minimize the variability of results compared with trabeculectomy [25], [26]. Visual function may deteriorate following surgery either as a result of complications of surgery or progression of disease. Although some studies reported decreased vision after surgery in both groups, there was no significant difference in vision recovery between both groups.

We previously published the first meta-analysis on the same subject, and a total of 8 articles were reviewed, including RCTs, a prospective series of cases, and retrospective studies [16]. However, we found no significant difference in terms of both qualified and complete operative success rates. While the current study was in progress, Chen et al [27] also reported a meta-analysis on the same topic. However, it appeared to be flawed because of included duplicated data and use of incorrect statistical methods [28]. Some specific points may explain the discrepant findings, which are considered as weakness points in the former analysis. First, non-RCTs and data from patients undergoing phacotrabeculectomy were included and evaluated in our previous meta-analysis, which may have increased the risk of biases in the analysis. The present meta-analysis included only RCTs and excluded trials in which phacotrabeculectomy occurred, using a wider range of clinically relevant outcome measures.Second, our previous study and meta-analyses by Chen et al did not separate the studies by the length of follow-up, which could have influenced the study results. Furthermore, there were several different criteria for normal IOP, such as IOP≤18 mm Hg, ≤20 mm Hg, ≤21 mm Hg, and so on. In present study, success was defined as a target endpoint of IOP≤18 mm Hg in all RCTs. Finally, the same study by Beltran-Agullo et al [7] and Patel et al [21] were included in the meta-analysis by Chen et al, but we only chose one paper. Of note, we added the latest RCT by Netland et al [8] involving 120 eyes, to increase the sample size and improve test performance. The pooled results showed that EX-PRESS was associated with significantly higher rates of complete operative success compared with Trabeculectomy. However, this should also be interprete with cautions. Only three of the four studies included this outcome. In addition, providers and outcome assessors were not masked to study group, providers may have added back medications differently for patients in one group versus another.

This meta-analysis has several limitations that should be taken into account when considering the results. First, the small numbers of cases per trial (range, 30–120) and in total give these analyses low power, especially for events with low incidence rates. Nevertheless, this meta-analysis provides more powerful evidence than individual reports alone. Second, patients were not stratified into high, medium, and low risk of filtration surgery failure subgroups; doing so may produce more interesting results. Third, most participants in the studies were Caucasian, and as there are reports of lower scarring and greater success rates of glaucoma surgery in Caucasians, these results may not be generalized to other races. Fourth, the examination of the rates of operative success and complications was based on pooled data from trials of different durations. Finally, the evaluator was not blinded to the procedure used in each trial. However, it is difficult to carry out a truly blind evaluation, as the type of surgery used is usually visible to the assessor.

Nonetheless, the present study provides additional interesting clues that may be useful for future research on this important topic. First, future studies need to focus on other important clinical endpoints (e.g., visual field and inflammation reactions) and biochemical indicators to better understand the benefits, mechanisms, and role of EX-PRESS in glaucoma [29]–[31]. In addition, quicker visual recovery or fewer postoperative visits were reported in 2 studies [7], [8]. However, we did not perform a meta-analysis because the reports lacked a uniform standard of measuring the restore speed of visual acuity. Finally, the period of follow-up is relatively short, and long-term data are essential for a robust assessment of the efficacy and complication rate of the EX-PRESS implant. Therefore, rigorous RCTs with long enough follow-up and large enough sample sizes are warranted to strengthen the body of evidence.

## Conclusions

In conclusion, our meta-analysis indicates that EX-PRESS is as effective as trabeculectomy in lowering IOP, medication reduction, and vision recovery. EX-PRESS associated with higher rates of complete operative success and fewer hyphema than with Trabeculectomy.However, conclusions drawn from our pooled results should be interpreted with caution because of the inherent limitations of the included studies.

## Supporting Information

Checklist S1 PRISMA checklist.
(DOC)
